# Acoustic accelerometry reveals diel activity patterns in premigratory Port Jackson sharks

**DOI:** 10.1002/ece3.5323

**Published:** 2019-07-27

**Authors:** Julianna Kadar, Monique Ladds, Johann Mourier, Joanna Day, Culum Brown

**Affiliations:** ^1^ Department of Biological Sciences Macquarie University Marsfield Australia; ^2^ Department of Conservation National Office Wellington New Zealand; ^3^ UMR MARBEC (IRD, Ifremer Univ. Montpellier, CNRS) Sète France; ^4^ Taronga Conservation Society Australia Mosman Australia

**Keywords:** accelerometer, activity pattern, diel cycle, migratory restlessness, Port Jackson shark, root mean square acceleration

## Abstract

Distinguishing the factors that influence activity within a species advances understanding of their behavior and ecology. Continuous observation in the marine environment is not feasible but biotelemetry devices provide an opportunity for detailed analysis of movements and activity patterns. This study investigated the detail that calibration of accelerometers measuring root mean square (RMS) acceleration with video footage can add to understanding the activity patterns of male and female Port Jackson sharks (*Heterodontus portusjacksoni*) in a captive environment. Linear regression was used to relate RMS acceleration output to time‐matched behavior captured on video to quantify diel activity patterns. To validate captive data, diel patterns from captive sharks were compared with diel movement data from free‐ranging sharks using passive acoustic tracking. The RMS acceleration data showed captive sharks exhibited nocturnal diel patterns peaking during the late evening before midnight and decreasing before sunrise. Correlation analysis revealed that captive animals displayed similar activity patterns to free‐ranging sharks. The timing of wild shark departures for migration in the late breeding season corresponded with elevated diel activity at night within the captive individuals, suggesting a form of migratory restlessness in captivity. By directly relating RMS acceleration output to activity level, we show that sex, time of day, and sex‐specific seasonal behavior all influenced activity levels. This study contributes to a growing body of evidence that RMS acceleration data are a promising method to determine activity patterns of cryptic marine animals and can provide more detailed information when validated in captivity.

## 1 INTRODUCTION

Activity patterns are a key component of animal fitness shaped by endogenous and exogenous factors that dictate behavior and determine species movement patterns (Helfman, 1986). Animals display movement patterns in response to endogenous circadian rhythm (Nelson & Johnson, 1970), to biotic factors such as prey availability, avoiding predators (Iwasa, 1982; Neilson & Perry, 1990), and reproduction, and to abiotic environmental factors such as light intensity (Appenzeller & Leggett, 1995; Bohl, 1979; Clark & Levy, 1988; Nelson et al., 1997) and temperature (Andrews et al., 2009; Sims et al., 2006).

Changes in light intensity and temperature, for example, are associated with changes in season and shifts from night to day (Cohen & Forward, 2016; McNamara, Mace, & Houston, 1987; Thiem et al., 2018). Both of these factors are key stimuli that drive patterns of animal behavior such as breeding and migration. Even though activity patterns are reasonably predictable over space and time, nocturnal and diurnal activity patterns can be subject to plasticity and may vary between sexes. Female catsharks, for example, remain inactive during daylight hours in shallower, warmer caves to avoid sexual harassment from males which are frequently active during the day and position themselves to intercept females upon their returns from nocturnal foraging trips into deeper waters (Wearmouth et al., 2012).

Continuing advances in technology have resulted in a golden age for biologging, greatly extending the limits of ecological research examining animal activity patterns (Wilmers et al., 2015). Biologgers are miniature devices attached to animals that transmit or log movement data and have been widely applied in marine habitats, particularly to elasmobranchs which are extremely difficult to study by traditional means (Hussey et al., 2015). Triaxial accelerometers are a relatively new type of sensor that can either log data at high frequencies (>100 Hz) or transmit low‐frequency data (5–10 Hz) to a receiver. Recording data at a lower frequency (i.e., lower resolution) and transmitting the data to a receiver means less detailed movement data are gathered but provides the advantages of longer recording duration without having to retrieve the tag. Triaxial accelerometers have been used to identify behavior patterns in elasmobranchs such as discriminating between periods of rest and activity (Whitney, Papastamatiou, Holland, & Lowe, 2007), identifying crepuscular fluctuations, and times of peak activity (Gleiss, Wright, Liebsch, Wilson, & Norman, 2013).

While conclusions can be drawn from raw accelerometer data alone (Kough, Jacobs, Gorsky, & Willink, 2018; Whitney, Lear, Gleiss, Payne, & White, 2018), captive studies are extremely valuable for validating accelerometer data and allow more detailed assessment of movement patterns and behavior (Brewster et al., 2018; Brownscombe, Gutowsky, Danylchuk, & Cooke, 2014; Goldstein, Dubofsky, & Spanier, 2015). Working in captivity allows close observation of the animal and matching behavior to accelerometer output, thereby enabling a more detailed calibration of accelerometer data with specific patterns of behavior. For example, a long‐term captive study on horseshoe crabs using accelerometers determined that the threshold for movement in relation to root mean square (RMS) acceleration was >0.1 m/s^2^ (Watson, Johnson, Whitworth, & Chabot, 2016). Similarly, calibration of video of American lobsters obtained in the laboratory with RMS acceleration allowed conversion of RMS to the distance travelled per unit of time and was also able to identify different intensities of movements such as burst events (Jury, Langley, Gutzler, Goldstein, & Watson, 2018).

Examination of animal behavior in captivity, however, may not be representative of behavior in the wild. Comparison of activity patterns from captive individuals to their wild counterparts may be necessary to ensure captive conditions are not influencing behavior. Activity budgets and endogenous rhythms have been shown to differ between captive and wild animals, though there are exceptions (Blasetti, Boitani, Riviello, & Visalberghi, 1988; Castro, Menezes, & Sousa Moreira, 2003; Höhn, Kronschnabl, & Gansloßer, 2000; Melfi & Feistner, 2002). Wild trout activity differed in captivity compared to when they were in their natal stream (Závorka, Aldvén, Näslund, Höjesjö, & Johnsson, 2015). In mammals, captive individuals tend to spend more time resting compared to those in the wild (Jaman & Huffman, 2008; Yamanashi & Hayashi, 2011). In contrast, many species of birds exhibit similar endogenous rhythms to free‐roaming counterparts characterized by increases in their activity levels at the time they would be migrating in the wild (Eikenaar, Klinner, Szostek, & Bairlein, 2014).

Sharks are key components of marine ecosystems, and through advances in technology, the movements of sharks can be examined in increasing detail. There are many knowledge gaps concerning elasmobranch behavior, especially during nocturnal or crepuscular periods when they are difficult to observe directly (Hammerschlag et al., 2017). In addition, there is a clear need to examine the movement patterns of small, temperate elasmobranchs more closely since they represent the majority of shark diversity and are rarely studied (Chapman, Feldheim, Papastamatiou, & Hueter, 2015). Port Jackson sharks, for example, play important roles in shaping their ecosystems by preying on echinoderms, which play key roles as ecosystem engineers on rocky reefs (Harrold & Reed, 1985). Thus, understanding the behavior of mesopredators can have ecosystem‐wide significance.

Port Jackson sharks are an ideal model for accelerometry owing to the fact that as a benthic, non‐obligate ventilator, it is easy to differentiate between phases of activity (i.e., swimming and resting) (also see Barnett, Payne, Semmens, & Fitzpatrick, 2016 study on whitetip reef sharks). They tend to have long periods of inactivity where they rest on the benthos punctuated by bursts of activity. Moreover, they are a very robust and adjust quickly to captivity. Port Jackson shark movement patterns have been examined using traditional approaches like SCUBA, scrutiny of catch records or the observation of captive individuals (McLaughlin & O'Gower, 1971; O'Gower, 1995; O'Gower & Nash, 1978; Powter & Gladstone, 2008), and have been described as primarily nocturnal; however, this pattern has not been formally quantified (O'Gower, 1995). Port Jackson sharks also migrate long distances and display sex‐specific migration patterns (Bass et al., 2017) suggesting that seasonal sex‐based differences in finer scale activity patterns may also occur. Capturing wild individuals and observing them in captivity for an extended period provides an opportunity for detailed observation of activity in relation to accelerometer output as well as comparing activity between captive and wild individuals. Moreover, calibration of behavior with accelerometer data in captivity facilitates interpretation of accelerometers deployed on wild sharks. Gaining a better understanding of Port Jackson shark activity patterns, in particular, the variation between sexes and seasons, is crucial to understanding the factors that shape their behavior in a rapidly changing environment.

Here, we tested the viability of an energy efficient accelerometer to examine the relationship between RMS acceleration measured from accelerometers and activity patterns in the Port Jackson shark captured on high‐definition video. Specifically, we aimed to (a) assess if RMS acceleration (recorded at 5Hz) could accurately depict predicted diel activity patterns; (b) examine if sex, time of day, and time of year influence Port Jackson shark activity patterns; and (c) compare the accelerometer activity pattern data from wild sharks held in captivity with activity patterns of free‐ranging sharks.

## 2 MATERIALS AND METHODS

Captive experiments were conducted with eight adult Port Jackson sharks at Taronga Zoo in Sydney, Australia, (−33°50′N, 151°14′E) from July–October in 2015 and 2016. Sharks were captured from Balmoral (33°49′S, 151°15′E) or Fairlight Beach (33°48′ S 151°16′E) in Sydney Harbor and transported to an outdoor enclosure at Taronga Zoo. The enclosure measured 17.8 × 8 m with a depth of 3.3 m and received constant water flow from Sydney Harbor that was mechanically filtered. To replicate caves that the sharks are found naturally inhabiting, three hides were placed on the floor of the enclosure. All individuals were measured for total length (TL) and weighed upon arrival. Sharks were allowed to acclimate to the enclosure for 1 week prior to experiments. During experimentation, two sharks were fitted with accelerometers at a time and allowed to swim freely in the enclosure. Temperature and water quality parameters were measured daily. The sharks were fed daily at 15:00 with squid, crab, or mussels ad libitum.

Three female and five male sharks participated in the experiments (Table 1). In two cases, data were obtained for less than 24 hr and were therefore excluded from the final analysis (ID 4763 and ID 4769), thus data were obtained for six sharks.

**Table 1:** Shark characteristics and deployment details for eight accelerometer deployments

Individual	Sex	TL (cm)	Attachment method	Attachment duration (days)	Month/Season	Year
5539	M	102	Dorsal	13.38	Sep–Oct (LB)	2015
5537	F	114	Dorsal	17.83	Oct–Nov (LB)	2015
5540	F	117	Dorsal	27.83	Sep–Oct (LB)	2015
4768	F	121	Harness	2.96	Aug (EB)	2016
4764	M	99	Harness	5.75	Aug (EB)	2016
4751	M	104	Harness	5.75	Aug (EB)	2016
4769	M	97	Harness	(15 hr)‐excluded	Aug (EB)	2016
4763	M	95	Harness	(18 hr)‐excluded	Aug (EB)	2016

Note

Two sharks were excluded from the study as a result of <24 hr of data collection.

Abbreviations: EB, early breeding season; LB, late breeding season; TL, total length.

### 2.1 Accelerometer attachment

**Figure 1:**
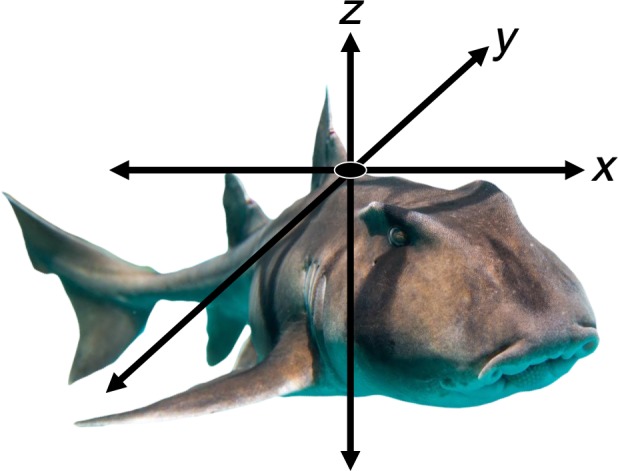
Representation of *x*, *y*, and *z* axes reflective of accelerometer attachment on each Port Jackson shark

Triaxial accelerometers measuring at 5Hz (Vemco V13AP, 90–150 s transmission delay) were attached to eight sharks using two methods—dorsal spinal needle (dorsal) and harness. For the dorsal attachment method, two stainless steel 14‐gauge Surflo IV surgical catheters (Terumo) were used to puncture the anterior end of the first dorsal spine. Suture thread, Prolene size 1 (Ethicon), was then threaded through the catheters and used to secure the accelerometer against the skin. The second method used a commercially available small‐animal harness (Petbarn) that was adapted to fit around the body of the sharks, over the first dorsal fin and behind the pectoral fins. The accelerometer was attached to the harness in close proximity to the dorsal spine. Both methods of attachment resulted in the same orientation of the three axes within the accelerometer (Figure 1). The accelerometers transmitted data acoustically to a receiver (Vemco, VR2W 69 kHz) within the enclosure.

The first 24 hr was excluded for each individual that underwent dorsal attachment due to elevated (Whitney et al., 2007) or irregular (Shipley et al., 2018) activity that can occur post‐tagging. Individuals that underwent harness attachment were not removed from the water, therefore no data were excluded.

### 2.2 Sampling

The accelerometers were preprogrammed to measure activity 8.3% of the time. These low‐resolution settings were chosen to mimic long‐term deployment methods. These methods are applicable in a wild setting where low‐resolution measurement at 5 Hz has been used for measurement of general activity levels (Watson et al., 2016). Every ~120 s the three axes were measured for 20 s and then converted into RMS (Equation 1) that summarized activity for the 20 s of measurement.

(1) $$\text{RMS}\;\left(m{\;s}^{\text{-2}}\right)=\frac{\sqrt{x^{2}+y^{2}+z^{2}}}{T}$$

where *x*, *y*, and *z* are the axes, and *T* is time. During the first year of experimentation in 2015, multiple sharks were already fitted with high powered acoustic tags (Vemco, V16‐6H) prior to adding the accelerometers. Due to the small size and concrete walls of the enclosure this resulted in both acoustic collisions and an overload of acoustic detections within the 69 kHz receiver. A collision model created by Vemco was used to illustrate the theoretic level at which the receivers were working. The model indicated that with 10 V16‐6H tags and 2 V13AP tags the receiver was working at a maximum detection probability of 40%–43% with an average detection period of ~300 s between consecutive detections for each tag.

This reduction in recordings did not affect overall analysis of activity patterns as recordings were relatively evenly spaced over a large number of days (14–29 days). In the second year of experiments, only two acoustic accelerometers were deployed in the enclosure at any one time resulting in 100% receiver recording rate (Table 2).

**Table 2:** Number of detections, attachment method, days recorded and detections for each shark within each year of experimentation

Individual	Year of trial	Attachment method	Average delay (seconds)	Days recorded	Total detections	Mean detections per hour
**5539**	2015	Dorsal	~300	13.38	1,846	5.75
**5537**	2015	Dorsal	~300	17.83	3,514	8.21
**5540**	2015	Dorsal	~300	27.83	3,770	5.64
**4768**	2016	Harness	~150	2.96	957	13.47
**4764**	2016	Harness	~150	5.75	1,804	13.07
**4751**	2016	Harness	~150	5.75	1,812	13.13

### 2.3 Video analysis

Video cameras (GoPro Hero4) were submerged in three locations in the enclosure to record movements of the tagged sharks during daylight hours. Twenty‐two videos ranging from 30 min to ~1 hr (h) (recorded at random times of the day from sunrise to sunset) were edited and exported at 50 fps using Final Cut Pro Software (Apple). The behavior of the sharks was scored minute by minute. If a shark (*n* = 3, male: 1; female: 2) was swimming for more than 10 s during the minute interval it received a score of 1 and if it was resting it received a 0 (as per Watson et al., 2016). The scoring methodology was applicable because preliminary observations suggested once a period of activity was initiated, the shark remained swimming for longer than 10 s. If the subject animal swam out of view of the cameras during a minute interval, the data were excluded from analysis. In order to calibrate the accelerometers, the video footage of the sharks was time‐matched with the accelerometry data. This was done by synchronizing the time on the accelerometer with the video. In this way, the accelerometer data could be converted to the proportion of time spent active based on the correlation with observed behavior. For each individual, we then calculated the mean proportion of time spent active for each hour over 24 hr.

### 2.4 Calculating proportion of time active

At the end of the observations, the acoustic receiver was removed from the enclosure and raw data were downloaded using VUE software (Vemco). For each individual, the mean RMS acceleration data were taken for each 1‐hr block corresponding to the behavior‐coded video footage. Binning the data into 1‐hr blocks was necessary because the sampling occurred at irregular intervals. 1‐hr blocks created a window long enough for multiple transmissions to be detected while still depicting detailed activity levels throughout the day. A simple linear regression was used to determine the relationship between time spent active and average RMS score.

### 2.5 Generating the linear mixed‐effects model

The 24 1 hr bins of RMS accelerometer values minimized serial autocorrelation within the activity data. The bins were transformed for normality using Box–Cox power transform (MASS package in R; Venables & Ripley, 2002). Multiple linear mixed‐effects models (LME) with restricted maximum likelihood estimation (REML) quantified the sources of variation that accounted for changes in activity levels (NLME package in R; Pinheiro, Bates, DebRoy, & Sarkar, 2014).

An information theoretic approach was applied to build candidate models. Candidate models consisted of individual shark ID and hour of the day as random effects to account for non‐independence of the data. Predictor variables included: sex, time of day (day/night), and time of year (early breeding season/late breeding season). Within the 1 hr bins, time of day was determined according to local times of sunrise and sunset. The fixed effects including interactions between all pairs of fixed effects were included in candidate models. The function dredge from package MuMIn was used to run all combinations of variables and interactions (Barton & Barton, 2013). Models were ranked using (AICc) (Table S1) (Sugiura, 1978). The lowest AICc was used to select the best model. The ability of random effects to strengthen the model was determined by the comparison of the final model to a null model in which random effects were excluded. Difference between the final and null models was determined by parametric bootstrap analysis applying exact likelihood ratio tests (RLRsim Package in R; Scheipl, Greven, & Küchenhoff, 2008).

To explain the amount of variance in activity patterns caused by the random effects of individual shark/hour of the day, marginal (fixed effect), and conditional (fixed and random effects) *R* ^2^ values were compared. Assumptions were tested by plotting predicted versus fitted residuals, QQ‐plots, Cleveland dot‐plots, and ACF plots to examine homoscedasticity, normality, homogeneity, and independence (Zuur & Ieno, 2016). To examine significant differences in mean active time between shark activity and the predictor variables, we used post hoc general linear hypothesis applying the Tukey method with the function glht from the multcomp package (Hothorn, Bretz, & Westfall, 2008). Analysis was completed in R (RStudio version 0.99.902; R Core Development Team, 2016). Values are reported as means (±*SD*), and significance was set at *α* = 0.05.

### 2.6 Wild activity patterns

Omnidirectional submersible ultrasonic receivers (miniSUR, Sonotronics Inc) were used to identify tagged individuals with acoustic transmitters. These receivers had a detection range set to shift every 5 min between 18 dB and 36 dB (corresponding to ~10 m and 60 m, respectively). The small spatial scale of this experiment reflects the relatively small range of Port Jackson sharks during breeding season (Bass et al., 2017). The choice to use miniSURs rather than VR2W longer range receivers was made to better record movements in this species during this low mobility period (Mourier, Bass, Guttridge, Day, & Brown, 2017). The receivers were able to record data sent from 12 acoustic transmitters (Vemco V16, 69 kHz, 90 s transmission delay) implanted into 39 (male: 25; female: 14) Port Jackson sharks (Bass et al., 2017). Eleven miniSURs (Sonotronics) were deployed in August–September 2016 on a breeding reef (Orion) in Jervis Bay, New South Wales (Figure S1). For this analysis, we restricted the recorded data to those recorded within a 10 m radius of each of the 11 receivers. Diel activity patterns were calculated based on the number of hourly movements between receivers. For this calculation, we counted each time a shark moved in and out of any receiver's detection range per hour which is indicative of activity levels. A Pearson's correlation was completed between captive and wild datasets to determine the strength of the linear relationship.

The frequency of movement model was constructed with the same approach as the captive activity model. Data were transformed (log(*x*) + 2) for normalization. Linear mixed‐effects models with REML estimated the factors influencing variation that dictated the frequency of wild shark movements (*n* = 12) (NLME package in R; Pinheiro et al., 2014). Random effects included shark ID and hour of the day, and fixed effects included sex, time of day, and sex * time of day interaction.

Consistent with the captive activity model, the final model was compared with a null model that had no random effects to assess the importance of the random effects. Parametric bootstrap analysis was used to determine the difference between the null and final models (5,000 simulations, Package in R: RLRsim; Scheipl et al., 2008). Examination of marginal and fixed *R* ^2^ values determined the variance within the wild shark movements that were caused by random effects. Assumptions were tested using the same methods as the captive model.

## 3 RESULTS

### 3.1 Predicting proportion of time active

**Figure 2:**
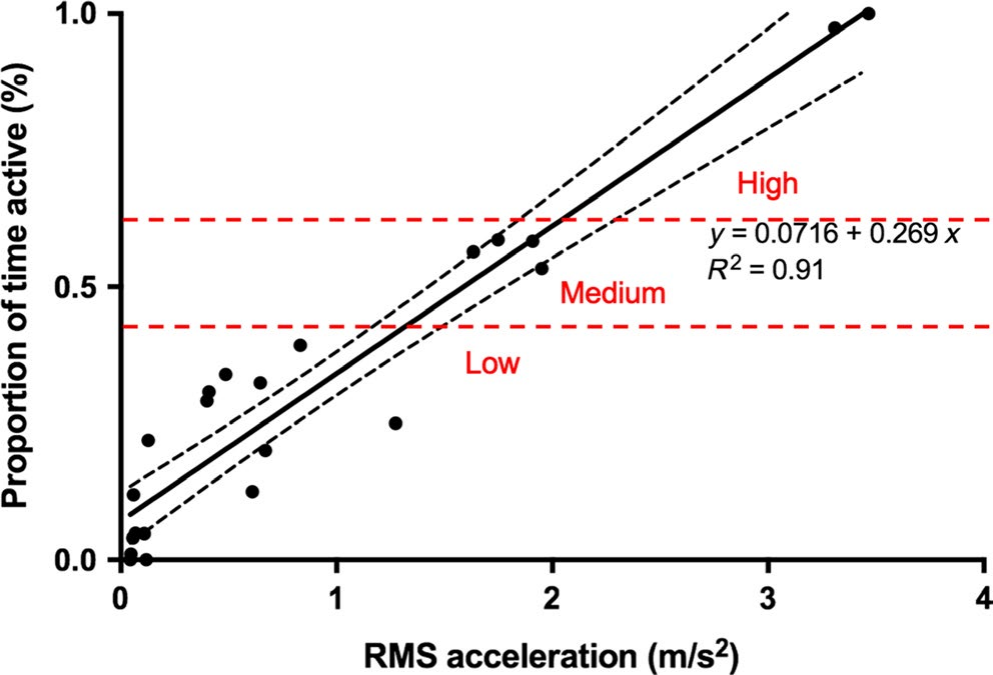
Simple linear regression comparing proportion of time sharks were active and RMS acceleration output with low, medium and high activity ranges denoted by dashed lines

Low‐resolution accelerometers allowed for the prediction of the amount of time each shark (*n* = 6) spent active per hour of the day (*R* ^2^ = 0.91; Figure 2). The linear regression resulting from video recording paired with accelerometry showed three distinct levels of activity as shown in Figure 2: (a) low level activity—the majority of time spent resting with occasional movement (16.97 ± 13.23% of time active); (b) medium level activity—swimming on the floor or foraging (56.67 ± 2.10%); and (c) high activity—swimming in the water column or vertically against the enclosure wall (99 ± 1.32%).

**Figure 3:**
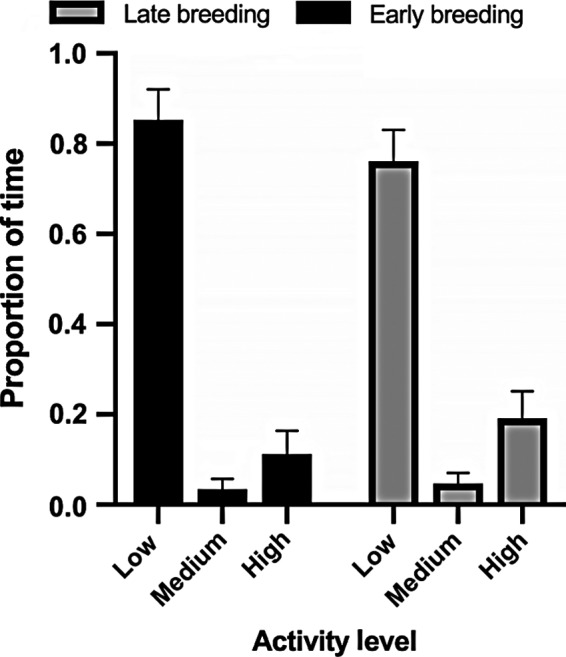
Proportion of time spent at three levels of activity for sharks in early and late breeding season. Low activity is 0 to <40% of time active, medium activity is ≥40 to ≤58% of time active, and high activity is >58 to 100% of time active

According to the distinct levels of activity derived from the linear regression, the proportion of time the sharks were active ranged from 8.5% to 78.7% per hour. On average, sharks were active 24 ± 11.8% of their time, either swimming on the enclosure floor, in the water column, foraging or vertical swimming. The sharks spent 81 ± 3.23% of their time within the low activity range, 4 ± 0.95% within medium activity range and 15 ± 2.70% within the high activity range. Sharks in late breeding season spent 8 ± 1.25% more time engaged in high activity behaviors (Figure 3).

**Figure 4:**
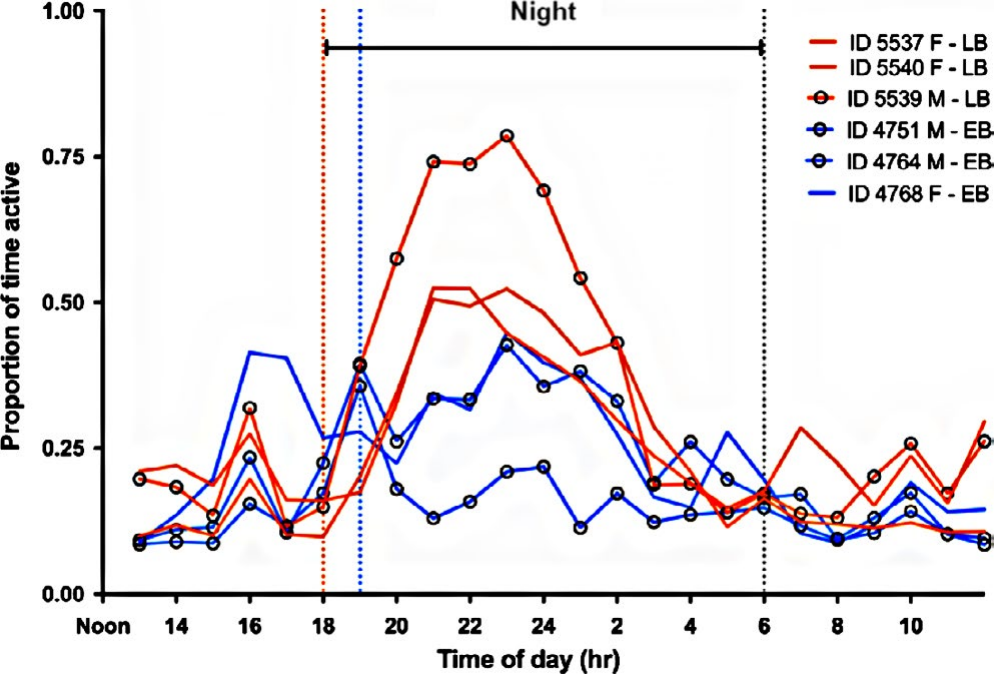
Mean diel level of activity of each individual shark in captivity (*n* = 6). Individuals studied early in the breeding season are depicted in blue and those examined late in the breeding season are in orange. Lines with open circle indicate males and lines without circles indicate females. Dashed lines indicate sunset and sunrise, two lines for sunset indicate shifting sunset times over early and late season. EB, early breeding season; F, female; LB, late breeding season; M, male

The diel pattern of the sharks in captivity consisted of an initial peak in activity from 15:00 to 19:00 (daily feeding occurred from 15:00–16:00), a main peak in activity from 18:00 to 23:00 and a decline of overall nocturnal activity from 00:00 to 04:00 (Figure 4). The period of least activity for all sharks ranged from 04:00 to 15:00 (i.e., early morning and daylight hours).

### 3.2 Factors influencing activity in captivity

The best fit activity model consisted of the fixed effects: time of day (day/night), sex, time of year (early/late breeding season), and sex * time of year interaction. Individual ID and hour of the day were included as random effects (AIC 292.56).

We found a significant relationship between RMS acceleration and time of day (Table 3). The sex of the sharks also had a significant effect on acceleration (Table 3). There was no effect of time of year, however the interaction between sex and time of year was significant (Table 3).

**Table 3:** Results of the LME for the relationship between RMS acceleration and explanatory factors

Factors	Intercept	*SE*	*t* value	*p* value
Sex	−0.43	0.14	3.18	0.0019
Time of day	1.05	0.17	6.00	<0.0001
Time of year	0.12	0.14	0.87	0.3874
Sex * time of year	0.74	0.19	3.87	0.0002

Abbreviations: *SE*, standard error; ^*^, interaction.

The strong interaction between sex and time of year was primarily driven by male behavior in October, which displayed the highest level of activity. The mean peak hour activity for this male was 0.786 ± 0.313 (ID 5539) while the next nearest individual's peak in activity was 0.523 ± 0.417 (ID 5540), both at 22:00. The most active male's (ID 5537) mean time spent active was 0.327 ± 0.221, which was substantially higher than the group mean (0.240 ± 0.118).

Further post hoc analysis of the interaction effect (sex * time of year) showed a significant difference between males and females in early breeding season (*p* = 0.009), and a strong contrast in activity levels between males in early versus late breeding season, with males being far more active in the late breeding season (*p* < 0.001). No significant difference was detected between females in early and late breeding season (*p* = 0.822) (Table S2).

**Figure 5:**
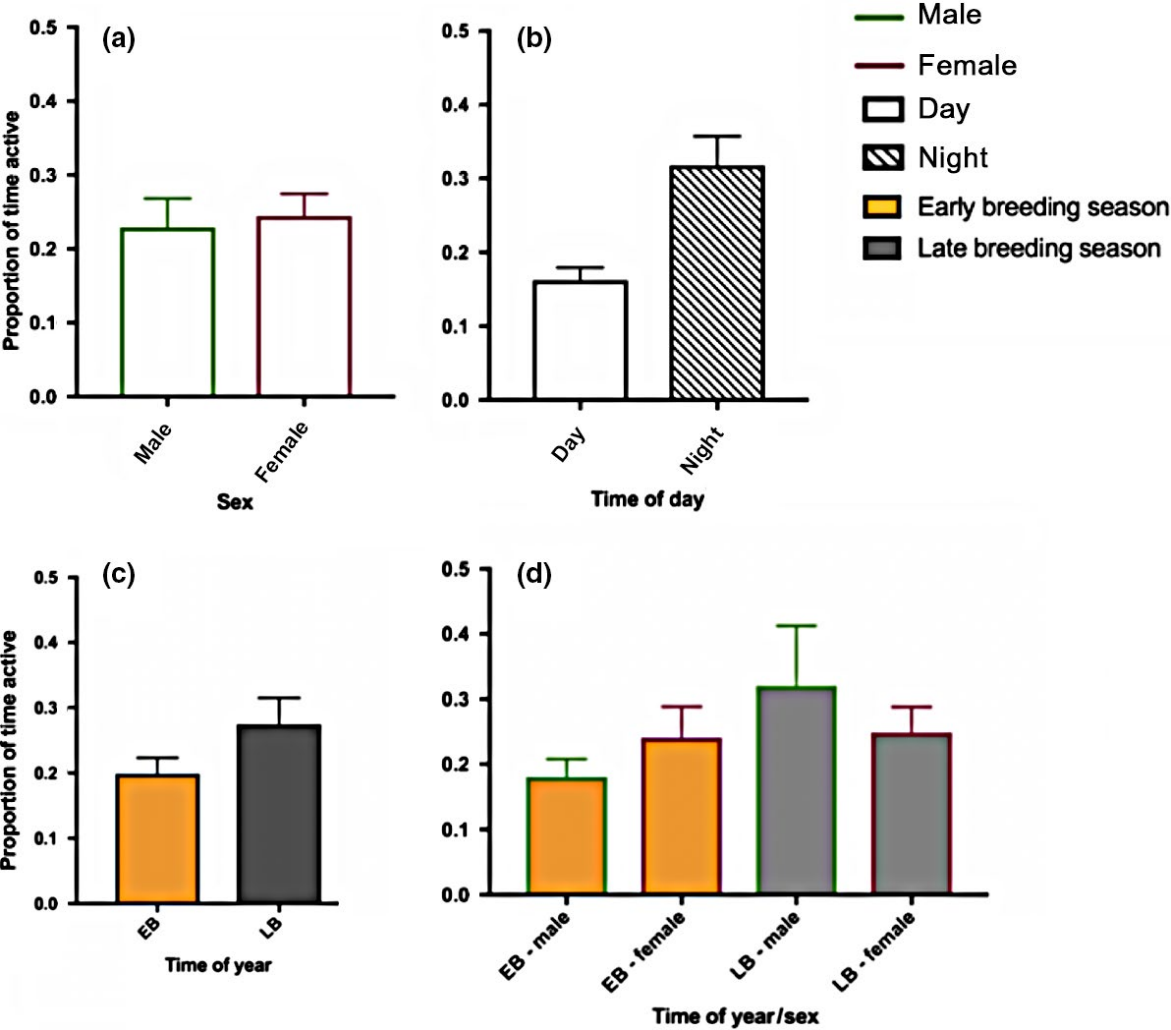
Mean (*SE*) proportion of time Port Jackson sharks spent active in captivity. (a) Males and females, (b) day and night, (c) early (EB) and late (LB) breeding season, (d) males and females within early and late breeding season

Parametric bootstrap analysis showed that including individual ID as a random effect improved model strength significantly (*p* = <0.001). Including hour of day also added greatly to model strength (*p* = <0.001), which was generated by the strong nocturnal activity pattern (Figure 4). Comparisons of marginal and conditional *R* ^2^ values for the final activity model (activity ~ time of day + time of year + sex + (time of year * sex)) showed high reliance on random effects within the model. Fixed and random effects explained 72.7% (conditional *R* ^2^) of the variance in activity and random effects alone accounted for close to half of that variance (marginal *R* ^2^ = 0.332) (Figure 5).

### 3.3 Wild activity patterns

**Figure 6:**
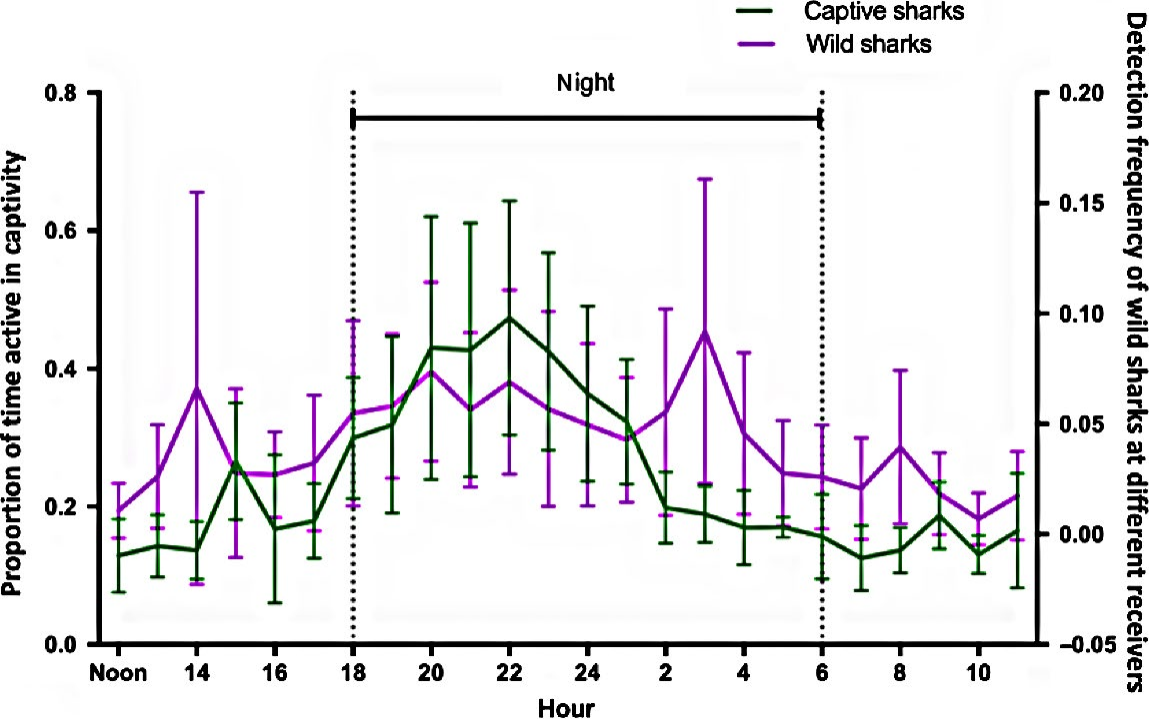
Mean diel level of activity of captive (green) and wild (purple) sharks (±*SD*). Dashed lines indicate sunset and sunrise

We found that the activity in wild sharks, as measured by the frequency of movements within a network of short‐range receivers covering their home reef, showed a moderate positive correlation (0.58) with activity patterns measured on captive sharks using accelerometers (Figure 6). Of the 39 sharks tagged, 12 remained within receiver range (male: 8; female: 4). The activity model for wild sharks consisted of the fixed effects: time of day (day/night), sex, and the sex * time of day interaction (AIC −1813.06). As per the captive model, Individual ID and hour of the day were included as random effects. There was neither sex effect nor an interaction between sex * time of day (*p* > 0.5), however time of day alone significantly influenced activity levels (*t* = 3.17, *p* = 0.004). Though the frequency of movement for wild sharks varied within a diel cycle, overall movement levels remained relatively low.

Parametric bootstrap analysis showed that while hour of the day improved model strength significantly (*p* = 0.058), individual ID was not significant (*p* = 0.310). Both the captive and wild shark activity models were improved by hour of the day, highlighting the strength of the nocturnal pattern. Comparing marginal and conditional *R* ^2^ for the wild activity model (activity ~ time of day + sex + (time of day * sex)) showed that 49.1% (marginal *R* ^2^) of the variance could be explained. Random effects composed ~15% of that variance (conditional *R* ^2^ = 0.1469).

**Figure 7:**
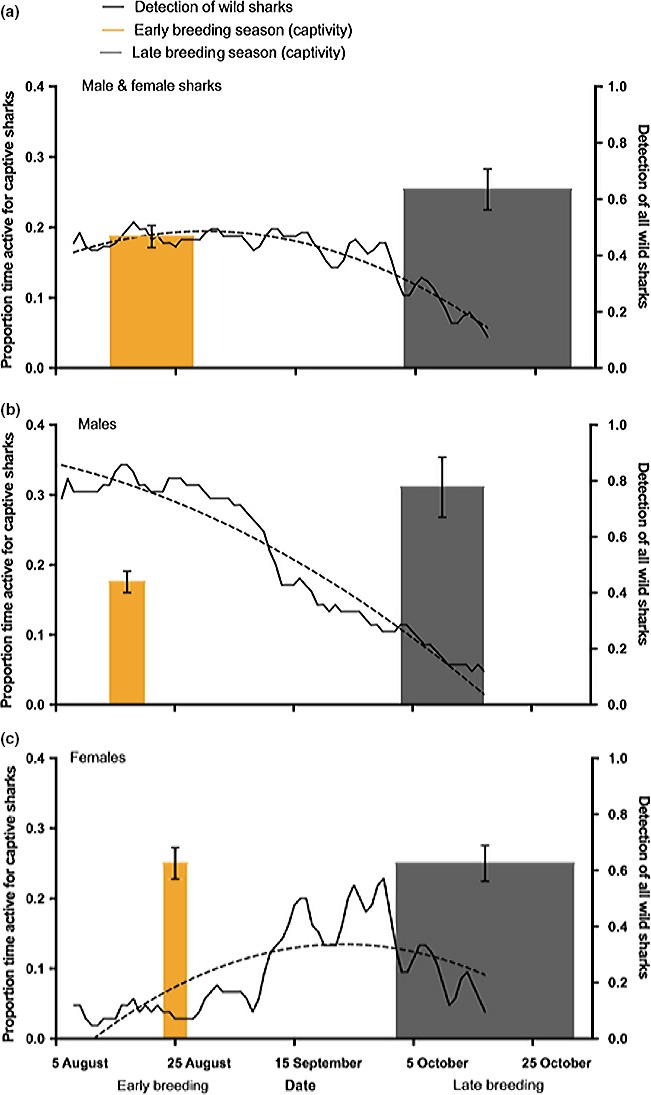
A comparison between detection of wild sharks (dashed line shown as polynomial best fit for moving average of raw detection) and captive shark activity levels (orange: early breeding season, gray: late breeding season). Male and female sharks are combined (a), then males (b) and females (c) separately. Bar width denotes months (August–October) as seen on *x*‐axis

We also found that the overall increase in activity patterns in captive sharks between the two periods corresponded to a drop in the proportion of sharks detected by the network of receivers (Figure 7). Captive male activity increased from early to late breeding season while female activity remained the same. During this time wild male detection rates markedly decreased compared to early breeding season.

## 4 DISCUSSION

Results from this study confirm that low‐resolution acoustic accelerometers attached to benthic elasmobranchs can return accurate information regarding their activity levels (Gleiss et al., 2017; Whitney, Pratt, Pratt, & Carrier, 2010). Port Jackson sharks generally exhibited nocturnal activity patterns, with most individuals peaking during late‐evening hours and exhibiting the lowest activity levels during early morning and daylight hours (Figure 4). There was little difference in activity of sharks during daytime for both the early and late breeding season, however sharks tended to increase in activity at night in late breeding season. Time of year influenced activity, but it varied with sex. The captive male in the late breeding season showed considerably higher activity levels possibly as an effect of migratory restlessness. The activity results obtained from accelerometers in the captive setting correlated moderately closely with those obtained by acoustic telemetry in the wild (Figure 6) suggesting the behavior exhibited in captivity mirrored that in the wild.

A review by Hammerschlag et al. (2017) found that the majority of elasmobranchs display increased movements during crepuscular periods rather than at night. Satellite telemetry shows sharks are more active during darker times (Comfort & Weng, 2015) and direct observations suggest sharks are foraging at this time (Fallows, Fallows, & Hammerschlag, 2016). Here, we found that Port Jackson shark activity rose in the hours just before and during sunset and declined before sunrise. Although artificial feeding times during daylight influenced natural activity in captive sharks, the majority of their activity took place during hours of darkness. They also spent the vast majority of their time in the low activity state. These patterns observed corroborate information from previous studies based on visual survey, tag and recapture, and acoustic tagging where juvenile Port Jackson sharks were found to spend the least amount of time active during the day and large amounts of time active during the night, but more time was spent inactive than active overall (Powter & Gladstone, 2009). Nelson and Johnson (1970) found similar patterns in horn sharks (*Heterodontus francisci*), a sister species, which remained inactive throughout the day while residing in caves and were more active at night. Adult Port Jackson sharks seem to socialize during the day while resting in large groups where they show preferences for particular individuals (Mourier et al., 2017). Port Jackson sharks may show greater preference for nocturnal activity than other elasmobranchs due to their specialized vision, which functions better at night (Hart, Lisney, & Collin, 2006; Schieber, Collin, & Hart, 2012). This characteristic aids in the occupation of sensory niches made possible through visual adaptations for darkness (Hueter, 1990).

Sharks in captivity displayed shifting activity patterns depending on the stage of their breeding season. Sharks observed in October, late in the breeding season, spent more time active and engaged in high activity level behavior than sharks early in the breeding season. Overall, males and females in October spent a higher proportion of their time active at night than the group earlier in the year. We also found differences in activity levels between sexes depending on the time of year. Given the small sample size of this study, the sex‐based results need to be interpreted cautiously as the observed differences could be an artifact of individual variation, especially in captive sharks. However, our models are based on large amounts of sampling per individual and suggest that sex may affect activity levels. The observed higher activity levels for males and lower activity for females in captivity may be reflective of contrasting reproductive strategies for both sexes. Males arrive to breeding grounds sooner and actively intercept females while females arrive later, stay later, and generally remain more sedentary, perhaps in an attempt to improve egg incubation and reduce egg mortality (Bass et al., 2017). Arrival and departure times to and from breeding grounds also reflect differences in environmental cues such as temperature and length of day that might influence movement patterns, though some endogenous rhythms may also influence behavior (Takemura, Rahman, & Park, 2010). Differences in activity between early and late breeding season are likely to be heavily influenced by increasing migratory pressure occurring in late breeding season, just before the scheduled southern migration from the breeding grounds (Bass et al., 2017).

High levels of activity in captivity, especially by males, late in the breeding season when they are due to depart for migration may be indicative of migratory restlessness. Migratory restlessness exhibits itself as increased activity in captive individuals during the time of departure for migration and can predict departure times in wild individuals (Eikenaar et al., 2014). This type of behavior has been extensively studied in birds and provides insight into the mechanisms that may be behind elevated activity patterns at this time (Liedvogel, Åkesson, & Bensch, 2011). Because Port Jackson sharks leave breeding grounds earlier than females (Bass et al., 2017), it is expected that captive males should show signs of migration restlessness earlier than females from October—November, when males in the wild begin migrating (Figure 7). Their departure from breeding sites in particular may be correlated with rising sea‐surface temperatures (J. Pini‐Fitzsimmons et al., unpublished data).

In the captive setting, both water temperature and day length could provide reliable cues to initiate migration. In salmon, for example, day length is a key cue for initiating migration and spawning (Liedvogel et al., 2011). Environmental and endogenous cues triggering migration may serve to optimize energetic costs. For example, movements made to avoid higher temperatures have been shown to play a role in mitigating energetic costs for male dogfish (Sims et al., 2006). There is also evidence that some Port Jackson sharks may use the East Australian current to facilitate their southern journey (Bass et al., 2017). That variation in northerly versus southerly activity patterns should be detectable using these methods. Thus, there is potential to use acoustic accelerometers to identify instances where environmental flow affects migrating sharks (Hays et al., 2016). Subsequently, the methods used here can be used to quantify the energetic costs and benefits of migration.

## 5 CONCLUSIONS

In summary, the use of accelerometers deployed on Port Jackson sharks in captivity showed correlations between observed behavior and acceleration data. These sharks were found to be nocturnal with sex‐specific seasonal activity patterns. The captive activity data matched the behavior observed in free‐ranging sharks moderately well as determined by acoustic telemetry. Increasing activity patterns in captivity corresponded to the timing of seasonal migration of Port Jackson sharks along the New South Wales coast. Future work should consider deploying accelerometers on wild sharks to examine their activity patterns during breeding, migration and nonmigratory seasons. Future work should also combine biologgers with acoustic telemetry to determine diel spatial ecology of individuals. By using a combination of methods which quantify both movement and shifts in position, home ranges, foraging strategies and patterns of space use become clearer (Legare, Skomal, & DeAngelis, 2018; Papastamatiou et al., 2018; Shipley et al., 2018). We suggest further study on free‐ranging Port Jackson sharks to collect information over a longer temporal scale particularly in regard to seasonal and sex‐specific movements across habitats and the routes taken during migration.

## CONFLICT OF INTEREST

None declared.

## AUTHOR CONTRIBUTIONS

CB conceived this study and the partnering of institutions by CB and JD made it possible. JK and JD collected and calibrated the captive data. ML led the direction of the captive data analysis and assisted JK in completing it. JM collected the wild data and assisted JK with wild data analysis. All authors contributed critically to an earlier manuscript written by JK and all authors gave final approval for publication.

## Data Availability

Data are available from the Dryad Digital Repository: https://doi:10.5061/dryad.hg4279m.
