# Supplementary material for: Acoustic accelerometry reveals diel activity patterns in premigratory Port Jackson sharks
Source: Ecol Evol. 2019 Jul 27;9(16):8933–44. doi: 10.1002/ece3.5323 (PMC6706188; doi:10.1002/ece3.5323)
Supplement: Supplementary file 1 [file ECE3-9-8933-s001.docx]

**SUPPORTING INFORMATION**

Figure S1. Map of receivers in Jervis Bay, NSW used to detect wild shark movement frequency and presence.

**LME model selection**

Table S1. Ten best fit models based on AICc score. TD: time of day, S: sex, TY: time of year, *: interaction.

+ The model with the lowest AICc score (activity ~ time of day + time of year + sex + (time of year*sex)) compared to the second scoring model (activity ~ time of day + time of year) had a difference in AICc score of 5.84. This resulted in a 0.0015 probability that the second-highest model minimized information loss.

| Model rank | Intercept | TD | S | TY | S * TY | TD * S | TD * TY | logLik | AICc |
| --- | --- | --- | --- | --- | --- | --- | --- | --- | --- |
| 1 | -1.31 | + | + | + | + |  |  | -137.28 | 293.90 |
| 2+ | -1.60 | + |  | + |  |  |  | -142.46 | 299.75 |
| 3 | -1.60 | + |  | + |  |  | + | -143.18 | 303.42 |
| 4 | -1.56 | + | + | + |  |  |  | -143.65 | 304.36 |
| 5 | -1.56 | + | + | + |  |  | + | -144.36 | 308.06 |
| 6 | -1.55 | + | + | + |  | + |  | -144.37 | 308.07 |
| 7 | -0.79 |  | + | + | + |  |  | -148.89 | 314.84 |
| 8 | -1.08 |  |  | + |  |  |  | -153.85 | 320.31 |
| 9 | -1.32 | + |  |  |  |  |  | -153.86 | 320.34 |
| 10 | -1.21 | + | + |  |  |  |  | -152.93 | 320.68 |

**Post hoc pairwise analysis**

Table S2. Post hoc pairwise comparison: Sex * Time of year (interaction). M: male, F: female, EB: early breeding season, LB: late breeding season.

| Contrasts | Estimate | SE | t value | P value |
| --- | --- | --- | --- | --- |
| F / EB – M / EB | 0.434 | 0.136 | 3.185 | 0.0099 |
| F / EB – F / LB | -0.118 | 0.136 | -0.868 | 0.8215 |
| F / EB – M / LB | -0.429 | 0.157 | -2.728 | 0.0365 |
| M / EB – F / LB | -0.552 | 0.111 | -4.954 | < .0001 |
| M / EB – M / LB | -0.863 | 0.136 | -6.329 | < .0001 |
| F / LB – M / LB | -0.311 | 0.136 | -2.284 | 0.1077 |
